# Genomic Features of *Cladobotryum dendroides,* Which Causes Cobweb Disease in Edible Mushrooms, and Identification of Genes Related to Pathogenicity and Mycoparasitism

**DOI:** 10.3390/pathogens9030232

**Published:** 2020-03-20

**Authors:** Rong Xu, Xiaochen Liu, Bing Peng, Peibin Liu, Zhuang Li, Yueting Dai, Shijun Xiao

**Affiliations:** 1 Internationally Cooperative Research Center of China for New Germplasm Breeding of Edible Mushroom, Jilin Agricultural University, Changchun 130118, China; xurong@jlau.edu.cn (R.X.); pengbing@jlau.edu.cn (B.P.);; 2 College of Plant Protection, Jilin Agricultural University, Changchun 130118, China; 3 Shandong Provincial Key Laboratory for Biology of Vegetable Diseases and Insect Pests, College of Plant Protection, Shandong Agricultural University, Tai’an 271018, China; zhuangli@sdau.edu.cn

**Keywords:** *Hypomyces rosellus*, *Lentinula edodes*, comparative genomics, evolution, pathogenicity, secretory protein

## Abstract

*Cladobotryum dendroides*, which causes cobweb disease in edible mushrooms, is one of the major fungal pathogens. Our previous studies focused on the genetic and morphological characterization of this fungus, as well as its pathogenicity and the identification of appropriate fungicides. However, little is known about the genome characters, pathogenic genes, and molecular pathogenic mechanisms of *C. dendroides*. Herein, we reported a high-quality de novo genomic sequence of *C. dendroides* and compared it with closely-related fungi. The assembled *C. dendroides* genome was 36.69 Mb, consisting of eight contigs, with an N50 of 4.76 Mb. This genome was similar in size to that of *C. protrusum*, and shared highly conserved syntenic blocks and a few inversions with *C. protrusum*. Phylogenetic analysis revealed that, within the Hypocreaceae, *Cladobotryum* was closer to *Mycogone* than to *Trichoderma*, which is consistent with phenotypic evidence. A significant number of the predicted expanded gene families were strongly associated with pathogenicity, virulence, and adaptation. Our findings will be instrumental for the understanding of fungi–fungi interactions, and for exploring efficient management strategies to control cobweb disease.

## 1. Introduction

Cobweb disease, together with dry bubble (*Lecanicillium fungicola*), green mold (*Trichoderma aggressivum*), and wet bubble (*Mycogone perniciosa*), are currently considered the four most serious diseases of edible mushrooms that are caused by parasitic fungi [1]. These fungal diseases pose threats to mushroom production, and cause economic losses in all mushroom-growing countries worldwide [2,3]. The incidence of these diseases is increasing at an alarming rate, causing serious damage to a variety of edible mushrooms, including *Agaricus bisporus* [4,5], *Pleurotus ostreatus* [6], *P. eryngii* [7], *Flammulina velutipes* [3], *Hypsizygus marmoreus* [8], and *Ganoderma sichuanensis* [9]. Cobweb disease is characterized by the rapid growth of abundant coarse mycelium over the affected mushrooms [10]. Typical symptoms of this disease are cottony fluffy white or yellowish to pink colonies on mushrooms, rapid colonization, and the covering of the host basidiomata by parasitic mycelia; these symptoms lead to host decay [11]. Several species of *Cladobotryum*, including *C. dendroides*, *C. protrusum*, *C. mycophilum*, *C. varium*, *C. multiseptatum*, and *C. verticillatum* have been identified as pathogens causing cobweb disease [10,12].

The sexual morph of the *Cladobotryum* is classified in the genus *Hypomyces* [13]. *C. dendroides*, the anamorph conidial state of *Hypomyces rosellus*, was first shown to cause cobweb disease in the edible mushroom *A. bisporus* [10]. Recently, *C. dendroides* was also found in *Lentinula edodes*, which seriously affects the quality and yield [14,15]. In previous studies, we have reported on several aspects of *C. dendroides*, including taxonomic classification, disease incidence, genetic and morphological characterization, pathogenicity, and fungicide screening [16]. However, the pathogenesis of this fungus at the molecular level remains largely unknown. Genome analysis will facilitate the identification of pathogenicity-related genes and the characterization of pathogen–mushroom interactions. However, sequenced genomes in this genus (*Hypomyces*/*Cladobotryum*) are scarce. To date, only the genomes of *C. protrusum* and *H. perniciosa* (the sexual morph of *M. perniciosa*) have been released [17,18]. *C. protrusum* is the only species of *Cladobotryum* for which a genome is available [17]. It is imperative to obtain additional genome data in order to rapidly identify pathogenic genes and characterize fungi–fungi interactions in the *Cladobotryum*.

The development of single-molecule real-time (SMRT) sequencing technology has tremendously increased the quality of pathogen genomic sequences [19]. Associated advances have improved the identification of pathogenicity-related genes and revealed the molecular mechanisms underlying pathogenesis. To date, several pathogenic genomes have been sequenced using Pacific Biosciences (PacBio) Sequel or the RSII platform [17,18,20]. With these available genomic sequences, it is possible to determine the evolutionary relationships among fungi [21,22,23,24], as well as to explore the evolution of nutritional mode. In addition, numerous gene clusters associated with pathogenicity have been identified, including secretory proteins, membrane transport proteins, pathogen–host interaction (PHI) genes, fungal virulence factors, and fungal G proteins [17,18]. In addition, some expanded or contracted orthologous, species-specific genes and positive selective genes, including protein kinases, serine peptidases, cell-wall proteins, carbohydrate-active enzymes (CAZymes), secondary metabolites (SMs), and P450, were shown to be involved in biological control activity, pathogenesis, and mycoparasitism [21,22,24].

In this study, we performed the whole-genome sequencing of *C. dendroides*, a mycoparasite causing cobweb disease in *L. edodes*. The objectives of our study were: (1) to present a high-quality reference genome for *C. dendroides*, and to explore the genomic features and genes related to pathogenicity and mycoparasitism; and (2) to conduct a comparative genome analysis within the genus *Cladobotryum* and estimate its evolution. Our assembled genome will further expand genomic datasets for comparative genomic analysis in Hypocreaceae and in other mycoparasitic fungi.

## 2. Results and Discussion

### 2.1. Genome Sequencing and Assembly

**Figure 1:**
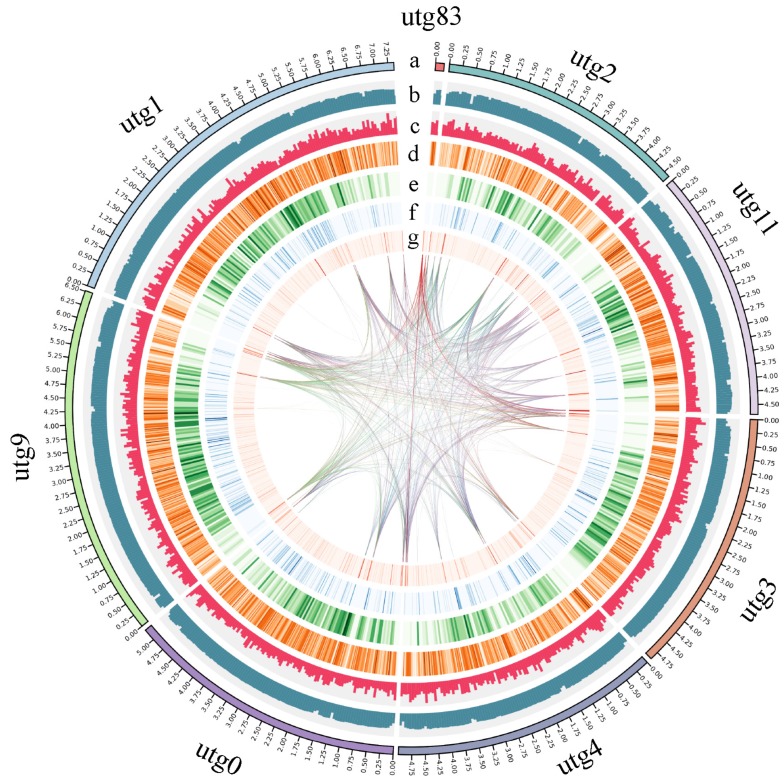
Genome map of *Cladobotryum dendroides.* Outer to inner concentric circles show (**a**) assembly contig number; (**b**) guanine-cytosine (GC) content; (**c**) sequence coverage; (**d**) gene density; (**e**) Benchmarking Universal Single-Copy Orthologs (BUSCO) estimates; (**f**) DNA repeat content; (**g**) long interspersed nuclear element (LINE), short interspersed element (SINE), and long terminal repeat (LTR) content.

We obtained genomic DNA from a single spore pure culture of *C. dendroides* (CCMJ2807) isolated from *L. edodes.* The genome was sequenced using the Sequel sequencing platform, and yielded 5.40 Gb of sequence data at 164× coverage. A total of 38.48 Mb of reads were assembled into eight contigs, with a contig N50 of 4.76 Mb, an N90 of 4.40 Mb, and a maximum span of 7.02 Mb. The size of the assembled genome was smaller than previously reported for *C. protrusum* (39.09 Mb) [17] and *M. perniciosa* (44.00 Mb) [18], but larger than other species within the Hypocreaceae, including *T. longibrachiatum* (32.24 Mb) [25], *T. reesei* (33.39 Mb) [26], and *Escovopsis weberi* (27.20 Mb) [27]. The guanine-cytosine (GC) content of the *C. dendroides* genome was 48.2%. The completeness of the assembled genome was evaluated by mapping the Benchmarking Universal Single-Copy Orthologs (BUSCO) set of 3725 fungal genes to the assembly. This analysis identified 3662 complete BUSCOs in the *C. dendroides* gene sets, which indicated a high-quality genome with an estimated completeness of 98.31% (Table 1 and Figure 1). Telomeres comprised of TTAGGG repeats are commonly identified at the molecular end of the linear chromosomes in filamentous fungi [28]. Among the eight contigs, four contigs (utg0, 1, 3, and 11) contained complete telomere-to-telomere structures, including remarkably regular repeat sequences ([T]TTAGGG). The remaining four contigs (utg2, 4, 9, and 83) could also be arranged into two complete telomere pairs, as each was comprised of one telomeric repeat. Consequently, there were six potential chromosomes in the *C. dendroides* genome, similar to related *Trichoderma* species, which have three to seven chromosomes [22]. These results indicated that our assembled genome was contiguous and sufficient for further analysis. Our new, high-quality, whole genome sequence of *C. dendroides* will provide insights into its evolution and pathogenic mechanisms.

### 2.2. Gene Prediction and Annotation

There were ~1.95 Mb of repetitive elements in the *C. dendroides* genome, which represented 5.07% of the total genome; this was consistent with previous findings in *C. protrusum* [17]. These repetitive elements comprised all of the major transposable element (TE) types, including DNA repeats, long interspersed nuclear elements (LINEs), short interspersed elements (SINEs), and long terminal repeats (LTRs). LTRs were the most abundant TE, representing 2.25% (0.86 Mb) of the genome, followed by LINEs (0.47%; 0.79 Mb) and DNA transposable elements (0.36%). Variations in genome size and structure, as well as in the numbers of predicted genes in the genome of a given species, are potentially closely associated with differences in repetitive DNA content; these differences may be due to natural selection, species lifestyle, and ecological niche [29]. *C. dendrodides* and *C. protrusum* have fewer repetitive elements, especially TEs, as compared *M. perniciosa* [17]. This might be because *M. perniciosa* has more efficient DNA removal mechanisms [18], and might explain the smaller size of the *M. perniciosa* genome. Genome size might be affected by host- and environment-associated adaptations as well as evolution [30,31]. We also performed a high-throughput screening of simple sequence repeat (SSR) loci, and identified 2520 SSR loci, including 817 dinucleotide repeats (DNRs), 644 trinucleotide repeats (TNRs), 782 tetranucleotide repeats (TTNRs), 203 pentanucleotide repeats (PNRs), and 74 hexanucleotide repeats (HNRs). These repeats included 454 types of motif sequences. The mean motif length was 27.72 bp, and the longest motif was 224 bp long, consisting of the TTNR “TTAT” repeated 56 times. The largest numbers of DNR, TNR, PNR, and HNR repeats were 47, 65, 26, and 12, respectively.

Gene predictions were performed using a combination of homology-based and de novo-based approaches. Using these approaches, we identified 9548 protein coding genes, with an average length of 1819.49 bp, in the *C. dendroides* genome. Across the identified genes, the average sizes of the exons and introns were 564.7 and 108.75 bp, respectively, which was similar to other species of Hypocreaceae [17,18]. We then functionally annotated these coding genes against seven typical databases, and found that 9369 (98.13%) genes had homologs in at least one database. Specifically, 9345 (99.74%) were homologous to known proteins in the National Center for Biotechnology Information (NCBI) non-redundant database (Nr); 4637 (49.49%) to known proteins in the Nucleotide Sequence Database (Nt); 5003 (53.40%) to known proteins in the Eukaryotic Clusters of Orthologous Groups (KOG) database; 7230 (77.17%) to known proteins in SwissProt; 4446 (47.45%) to known proteins in the Gene Ontology (GO) database; 5203 (55.53%) to known proteins in the Kyoto Encyclopedia of Genes and Genomes (KEGG); and 5381 (57.43%) to known proteins in Pfam. The total length of the non-coding RNA (ncRNA) was 34,626 bp, accounting for 0.09% of the assembled genome. The ncRNA contained 225 transfer RNAs (tRNAs), 26 small nuclear RNAs (snRNAs), and 56 ribosome RNAs (rRNAs).

### 2.3. Identification of Mating-Type Idiomorphs

We identified two previously-characterized MAT genes (MAT1-1-2 and MAT1-1-3) grouped together in the genome at a single locus (contig utg0) (Supplementary Figure S1). The MAT 1-2 idiomorph was not found in *C. dendroides*, incongruent with results in *C. protrusum* and *M. perniciosa*, but consistent with results in *Macrophomina phaseolina*, *T. harzianum*, and *T. longibrachiatum* [21,22]. In addition, the genes adjacent to the MAT genes were all located upstream on this locus, including complex I intermediate-associated protein 30 (CIA30), anaphase-promoting complex (APC5), cytochrome C oxidase subunit VIa (Cox), and DNA lyase (APN2). Notably, the flanking genes in *C. dendroides* were the same as those in *M. perniciosa* (contig utg16) and were oriented in the same direction [18]. The identification of these mating-type structures in the *C. dendroides* genome provides some insights into the sexual lifestyle of this organism.

### 2.4. CAZymes Analysis

**Figure 2:**
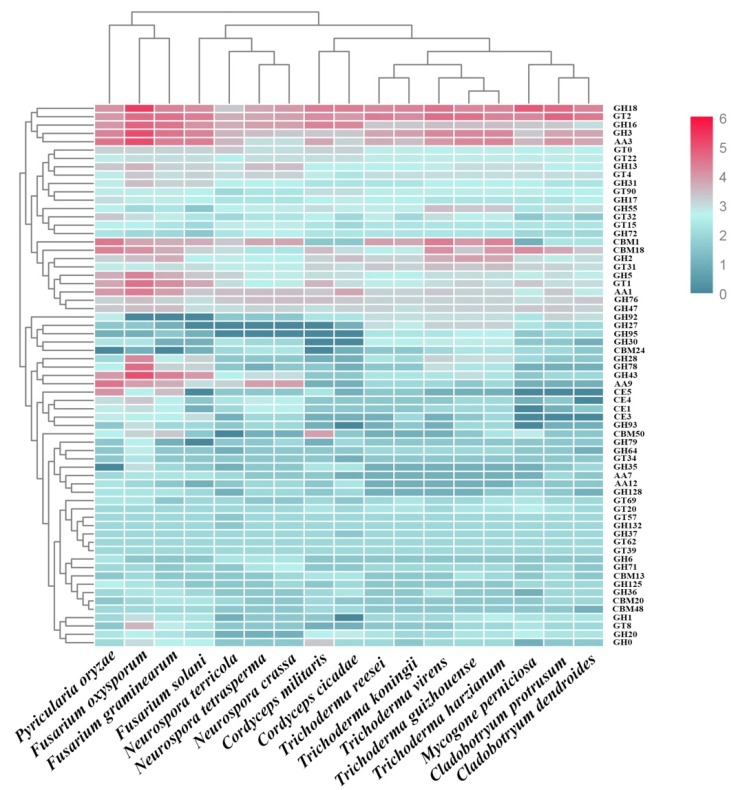
Comparative analysis of the genes encoding carbohydrate-active enzymes (CAZymes) in *Cladobotryum dendroides* and 16 other fungi. Over-represented (6 to 0) values are depicted as Z-scores for each row in the heatmap.

At the beginning of infection, pathogens primarily use CAZymes to destroy the polysaccharide component of the host cell wall [32]. We identified 327 *C. dendroides* genes across the six categories of CAZymes. We found that 163 genes, representing the largest proportion of the genome, encoded glycoside hydrolases (GHs), 103 genes encoded glycosyl transferases (GTs), 27 genes encoded carbohydrate-binding modules (CBMs), and 27 genes encoded auxiliary activities (AAs). The carbohydrate esterases (CEs; 4 genes) and polysaccharide lyases (PLs; 3 genes) were poorly represented in the genome. *C. dendroides* had fewer CAZymes than several other species in the Hypocreaceae: *T. harzianum* (439), *T. virens* (434), *T. guizhouense* (423), *C. protrusum* (364), *T. reesei* (360), *T. koningii* (353), and *M. perniciosa* (338) (Figure 2).

Across the three pathogens (*C. dendroides*, *C. protrusum*, and *M. perniciosa*), most genes encoded GH and GT enzymes, which might be used to degrade the host cell barrier during the fungi–fungi infection process. Most of the differences between the two *Cladobotryum* and three *Trichoderma* (*T. harzianum*, *T. virens*, and *T. guizhouense)* genomes were due to the high copy number of GH and CBM families in these *Trichoderma* genomes. Most chitinase- and cellulose-degrading enzymes are categorized within GH classes. The ability of *C. dendroides* to decompose chitin is suggested by the abundance of genes that belongs to the GH families. Of the GH families, GH18 is encoded by the most genes (19), followed by GH3 (13). GH18 contains the class III and V chitinases. The abundance of GH18 and GH3 was consistent with the efficient degradation of chitinase, cellulose, and hemicellulose [33], and suggested that these enzymes might play a role in the *C. dendroides* genome. In addition, we found that the *C. dendroide* genome contained a large number of CBMs, which occur as fusions to GHs, CEs, or AAs. CBM18 is most abundant in the CBM family in *C. dendroides*: approximately nine residues in modules. These modules were attached to a number of chitinase catalytic domains. Chitin-binding functions have been demonstrated in many cases [34]. Our results suggested that *C. dendroides* might destroy the host cell wall by secreting a large amount of chitinase during infection. The CBM1 and CBM50 binding domains have been previously described in the order Hypocreales [22,35]. We found additional CBMs that putatively bind to starch, xylanase, fructans, cellulose, and glucans. This implied that *C. dendroides* makes full use of these domains, which might result in the faster and more competitive degradation of the respective polymers.

AAs, which are involved in lignin degradation, also account for a large proportion of the CAZymes in *C. dendroides* (27). The relative abundances of the AA families were larger than *M. perniciosa* (22) [18], but fewer than *C. protrusum* (34) [17]. Of the AA enzymes, AA3 was the most abundant family (13), followed by AA1 (6), AA7 (2), AA12 (2), and AA9 (1). A previous study showed that the AA3_2 subfamily (including both aryl alcohol oxidase and glucose 1-oxidase), combined with AA9 lytic polysaccharide monooxygenases (LPMOs), can catalyze the efficient cleavage of cellulose [36]. We also detected 11 AA3_2 subfamilies in *C. dendroides*, compared with 14 in *C. protrusum* [17]. The abundant GT2 (21 genes) also suggested that *C. dendroides* has abundant enzymes associated with cellulose synthase and chitin synthase.

### 2.5. Analysis of Secondary Metabolites (SMs) 

SM are important virulence determinants in several fungal pathogens, and are also essential for stress tolerance and cellular signaling [37]. We identified 116 SM genes in *C. dendroides* using antiSMASH, compared with 143 in *C. protrusum* [17]. The SM genes in *C. dendroides* included 48 type 1 polyketide synthases (T1PKS), 36 non-ribosomal polypeptide synthetases (NRPS), 17 terpene synthases (TS), nine NRPS-like genes, three fungal-RiPPs, two siderophores, and one indole encoding gene clusters. More PKSs and NRPSs were identified in *C. dendroides* than in *C. protrusum* [17], *M. perniciosa* [18], and *Trichoderma* spp. [22]. In addition, we used Natural Product Domain Seeker (NaPDoS) analysis to search for genes encoding secondary metabolites in *C. dendroides.* A total of 100 genes encoding SMs were identified, including various pathway products such as HC-toxin, cyclo, compl, ituri, prist, tyroc, liche, bacit, act, cdaps, surfa, and micro. These results suggested that *C. dendroides* might synthetize many essential biologically-active compounds.

### 2.6. Prediction and Analysis of Pathogenicity-Related Genes

Pathogens can secrete many proteins that support the colonization of the host surface during infection [38]. To identify potential protein-coding genes related to pathogenicity and virulence in the *C. dendroides* genome, BLAST searches against the pathogen–host interaction database (PHI) and the database of fungal virulence factors (DFVF) were performed. We predicted 419 and 153 genes with ≥70% identity in the PHI and DFVF, respectively, representing 4.39% and 1.60%, respectively, of the predicted total genes (Supplementary Tables S1 and S2). Additionally, 76 genes were identified in both databases. Of the PHI genes, reduced virulence (203, 48.45%) was the most abundant followed by unaffected pathogenicity (139, 33.17%), loss of pathogenicity (28, 6.68%), lethal (40, 9.55%), increased virulence (hypervirulence) (5, 1.19%), chemistry target (3, 0.72%), and enhanced antagonism (1, 0.24%). No effectors (plant avirulence determinants) were found with ≥70% identity.

Using SECRETOOL, 371 candidate secretory proteins were predicted. Remarkably, 287 peptidase proteins with an identity ≥70% were identified against the peptidase database (MEROPS) database (Supplementary Table S3). Among these secretory proteins, seven homologs were identified in MEROPS, and three homologs were identified in PHI. Fungal effectors can play a vital molecular role in fungus–plant communication, targeting and regulating the phytohormone signaling of hosts by changing or operating them [39]. A set of 400 putative effector proteins were predicted using the filtered set SignalP (identify ≥70%), out of which seven were unique genes. Previous studies have predicted several effector proteins in *C. protrusum* [17] and *M. perniciosa* [18]. Pathogens may optimize their own effector sets to adapt to hosts [40]. Overall, these fungal effectors in *C. dendroides* might lead to the adaptation of pathogens with a broad host range to a specific host over time [41]. We also identified 336 (3.52%), 175 (1.83%), and 48 (0.50%) genes encoding for cytochrome P450 (CYP), major facilitator superfamily (MFS) transporters (Pfam domain assignment), and ATP-binding cassette (ABC) transporters, respectively.

### 2.7. Comparative and Evolutionary Analysis

**Figure 3:**
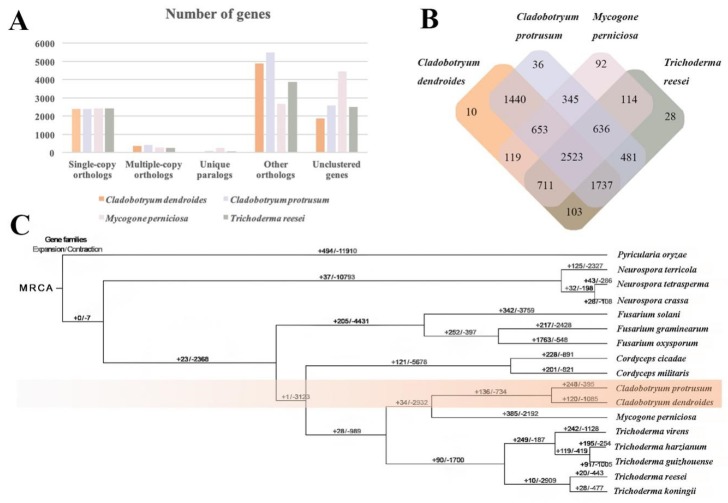
Genomic analyses, comparing *Cladobotryum dendroides* with other fungi. (**A**) Orthologous genes in the genomes of *C. dendroides*, *C. protrusum*, *Mycogone perniciosa*, and *Trichoderma reesei*. (**B**) Unique and shared gene families among the four genomes. Overlaps indicate shared gene families. (**C**) Phylogenetic tree of *C. dendroides* and 16 other fungal species, constructed using the maximum-likelihood method. MRCA, most recent common ancestor.

To explore gene family evolution in *C. dendroides*, we aligned its coding sequences (CDSs) with those of *C. protrusum* and 15 other representative fungi downloaded from the NCBI: *M. perniciosa* [18], *Pyricularia oryzae* [42], five *Trichoderma* (*T. virens* [43], *T. reesei* [26], *T. koningii* (https://www.ncbi.nlm.nih.gov/genome/?term=Trichoderma++koningii), *T. guizhouense* (https://www.ncbi.nlm.nih.gov/genome/?term=Trichoderma++guizhouense), and *T. harzianum* [44]), three *Neurospora* (*N. terricola* [45], *N. tetrasperma* [46], and *N. crassa* [47]), two *Cordyceps* (*Cor. militaris* [48] and *Cor. Cicadae* [49]), and three *Fusarium* (*F. solani* (https://www.ncbi.nlm.nih.gov/genome/?term=Fusarium+solani)*, F. oxysporum* [50], and *F. graminearum* [51]). A total of 8957 ortholog families (groups), comprising 10,983 proteins, were produced, as well as 166 orphans that showed no homology with any other proteins in the dataset. A total of 7684 and 8418 groups (in *C. dendroides* and *C. protrusum,* respectively) had homologs in the other fungi tested, of which about 2523 were conserved across all of the compared genomes (Figure 3A). *C. dendroides* and *C. protrusum* shared the most orthologous gene families (6356). *C. dendrodides* shared the fewest unique orthologous gene families (10) with *C. protrusum* (36), *M. perniciosa* (92), and *T. reeseirep* (resentative of *Trichoderma*, 28) (Figure 3B). These unique genes might be relevant to the resistance and adaption abilities of *C. dendroides*.

We next phylogenomically analyzed 2287 single copy orthologs that were conserved across all of the fungi analyzed (Figure 3C). Our phylogeny of the 17 species indicated that *Cladobotryum*, *Mycogone*, and *Trichoderma* were distantly-related genera within the family Hypocreaceae; this was consistent with previous reports [18,22,43]. Furthermore, *Cladobotryum* was more genetically similar to *Mycogone* than to *Trichoderma*, which was consistent with morphological taxonomy, traditional classification, and life history. Our phylogeny also recovered *C. dendroides* and *C. protrusum* in a monophyletic clade, and showed that these species diverged relatively recently. Based on phylogenetic placement, we also inferred that, within the *Trichoderma* clade, subgroup I (consisting of *T. virens*, *T. harzianum*, and *T. guizhouense*) evolved independently of subgroup II (*T. reesei* and *T. koningii*), and occurred species differentiation. This conclusion was consistent with that of Kubicek et al. [43]. These results indicated that our phylogeny tree accurately reflected evolutionary relationships.

We found that gene family contraction in *C. dendrodides* was more common that gene family expansion. A total of 120 gene families in *C. dendroides* expanded during evolution, fewer than in *C. protrusum* (248) and *M. perniciosa* (385) (Figure 3C). Functional analyses showed that the significant expansion of gene families in *C. dendroides* were mainly associated with the major facilitator superfamily (MFS), ankyrin repeats (three copies), and NACHT domains (*P* < 0.05). MFS transporter proteins allow the uptake of sugars to permit growth on varied substrates [52]. MFS gene families with three duplications were expanded in *C. dendrodides*, providing this fungus with the versatile ability to utilize sugars for growth. NACHT domains and ankyrin repeats are two kinds of heterokaryon incompatibility protein (HET) related domains. NACHT domains are predicted NTPases, which play a role in programmed cell death (PCD); PCD might be ancient, preceding the radiation of animals and fungi [53,54]. The ankyrin proteins modulate protein–protein interactions among HET proteins [55]. HET and related genes are essential for genetic information transfer in *Pyrenochaeta lycopersici* [55]. Interestingly, MFS transporters and ankyrin repeats were also expanded in *M. perniciosa* and *Trichoderma* species [18,22]. Furthermore, in order to adapt to unfavorable conditions and to host defense mechanisms, pathogens must generate variation [55]. Therefore, this expansion of multiple gene families in *C. dendrodides* might play a significant role in the pathogenicity of this species, as well as its ability to effectively infect macrofungi, to tolerate varied climates, and to adapt to different host lifestyles [56]. We identified 2.75-fold more contracted gene families in *C. dendroides* (1085) than have been found in *C. protrusum* (395) [17], but twice as many were identified in *M. perniciosa* (2192) [18]. These contracted gene families may serve to decrease the host range of *Cladobotryum* and *Mycogone* compared to *Trichoderma* species [18]. In addition, all of the noticeably contracted gene families were predicated to be P450 members (CYP5150A2, CYP5151A1, and CYP620H1) (*P* < 0.05). We identified 278 significantly positively selected genes in *C. dendroides* (*P* < 0.01), which were mainly related to transport and catabolism, the metabolism of cofactors and vitamins, the immune system, signal transduction, sorting and degradation, and cell growth and death. These genes might play a crucial role in the adaptation of *C. dendroides* to harsh environments, and in its mycoparasitic lifestyle.

**Figure 4:**
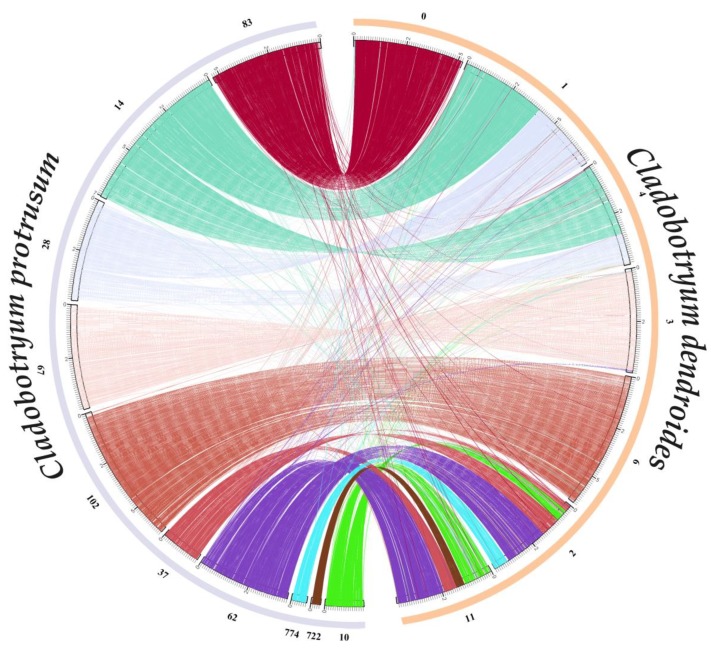
The genome collinearity analysis of the *Cladobotryum dendroides* and *C. protrusum* based on protein-coding genes.

Whole-genome collinearity analyses for *C. dendroides* and *C. protrusum* were then performed (Figure 4). Most of the contigs in *C. dendroides* were highly conserved syntenic blocks shared with *C. protrusum*. For example, contig9 and contig0 from *C. dendroides* corresponded with contig102 and contig83, respectively, from *C. protrusum*. We identified a few inversions and rearrangements between the homologous regions of the two genomes. For example, we observed a large inversion between contig3 from *C. dendroides* and contig67 from *C. protrusum*, as well as rearrangements in contig1 and contig4 from *C. dendroides* and contig14 and contig28 of *C. protrusum*. This suggested that a set of fusions or breakages might have arisen among the chromosomes of *Cladobotryum* species during their long evolutionary history. Notably, we observed an inversion between the middle region of contig9 from *C. dendroides* (145 genes) and the middle part of contig83 from *C. protrusum* (150 genes). Functional analyses showed that the 145 *C. dendroides* genes were mainly associated with GH18 (chitinase), GH35 (beta-galactosidase), serine/threonine-protein kinase, MFS, P450, and transcription factors. Additional genes located in other inversions and rearranged regions were related to amidase, glutathione S-transferase, GH3, GH16, signal peptidases, and zinc-finger proteins (C2H2 type). This genomic diversity might play a crucial role in the morphological formation, lifestyle, adaptation, and resistance of these two species.

## 3. Materials and Methods

### 3.1. Culture Conditions and Genomic DNA Extraction

A single virulent *C. dendroides* spore was isolated from a specimen of *L. edodes* infected with symptomatic cobweb disease in Qingyuan, Zhejiang Province (China). This strain was defined as CCMJ 2808, and it was used for whole genome sequencing. We confirmed the identification of this strain as *C. dendroides* using morphological characters, molecular analysis of ITS DNA sequences, and artificial inoculation [15]. After culturing strain CCMJ 2808 in potato dextrose agar (PDA) at 25 ºC in the dark for three days, mycelial mats of pure isolates were harvested and frozen using liquid nitrogen. Genomic DNA extraction was performed using Nuclean plant genomic DNA kits (CWBIO, Beijing, China), following the manufacturer’s protocol. We measured DNA integrity, purity, and concentration using 0.6% agarose gel, a Nanodrop 2000 (Thermo Fischer Scientific, Life Technologies, USA), and a Qubit 3.0 (Thermo Fischer Scientific, Life Technologies, USA), respectively. Strain CCMJ 2808 was maintained in the Engineering Research Center of Chinese Ministry of Education for Edible and Medicinal Fungi (ERCCMEEMF, Changchun, China).

### 3.2. Genome Sequencing and Assembly

We constructed 20-kb libraries for *C. dendroides*, and the genome was sequenced with a PacBio Sequel long-read sequencing platform [17,18,23,57]. To redress mismatch rate and increase sequencing read lengths, we performed next generation sequencing. SMARTdenovo (https://github.com/ruanjue/smartdenovo) was used for the de novo assembly of the genome. BUSCOs [58] were used to assess the completeness of the genome assembly. The assembled genome of *C. dendroides* was submitted to the NCBI database and deposited with the accession number WWCI01000000.

### 3.3. Gene Prediction and Genome Annotation

Repeat elements (including SSRs) and non-coding genes were predicted as described previously [17,18]. Coding genes were predicted using a combination of sequence homology and de novo prediction. The homology approach was based on representative reference genomes, including those of *Cor. militaris* [48], *T. reesei* [26], *C. protrusum* [17], *F. oxysporum* [50], and *F. graminearum* [51]. De novo predictions were performed using Augustus [59] and GlimmerHMM [60]. Finally, MAKER2 [61] was used to combine the gene models generated by both de novo prediction and protein homology matching. The predicted genes were used for subsequent analyses. The predicted protein-coding genes were functionally annotated against seven databases, with a cutoff e-value of 1 × 10^−5^: NCBI Nr, Nt, KOG [62], SwissProt [63], GO [64], KEGG [65], and Pfam [66]. The presence of mating-type genes in *C. dendroides* was confirmed by homologous alignment with mating-type genes and flanking sequences from the order Hypocreales. Biological sequences 1.0 was used to draw the gene structures [67].

### 3.4. Secretome Analysis and Pathogenicity-Related Genes

Secretory signal peptides were analyzed using the web-based analysis pipeline SECRETOOL, which is specifically designed to screen and identify putative secretome in fungi [68]. Protease candidates were screened by searching secretory proteins against the MEROPS database [69]. Pathogenic genes were identified using the PHI (http://www.phi-base.org/) [70] and DFVF (http://sysbio.unl.edu/DFVF/Download.php) [71] databases. Effector candidates were predicted in combination with the SignalP 5.0 server (http://www.cbs.dtu.dk/services/SignalP-5.0/). Then, virulence-associated genes, Cytochrome P450s, CAZymes, and SMs were predicted as described by Sossah et al. (2019) [17].

### 3.5. Orthological, Phylogenetic, Evolutionary, and Whole-Genome Collinearity Analyses

OrthoMCL (v.2.0.9) [72] was used to identify the orthologous gene families based on the CDSs of *C. dendroides*, *C. protrusum*, *M. perniciosa*, and 14 other selected genomes downloaded from the NCBI. The shared single-copy genes were screened and aligned using Clustal omega [73]. To construct a genome-based phylogenetic tree, we used the maximum likelihood (ML) algorithm in RAxML [74], with *P. oryzae* as the outgroup. Computational Analysis of Gene Family Evolution (CAFE) 3.1 [75] was used to predict the expansion and contraction of the orthologous gene families; those with a *P-*value < 0.05 were considered to be significant candidates. Positively selected genes were identified using the branch-site model of the CodeML tool in PAML [76]. The whole-genome collinearity of *C. dendrodides* and *C. protrusum* was analyzed using the Python version of MCscan, with default parameters (https://github.com/tanghaibao/jcvi/wiki/MCscan- (Python- version)).

## 4. Conclusions

In this study, we obtained a high-quality genome sequence of *C. dendroides*, a causal agent of cobweb disease on cultivated edible mushrooms, using the PacBio Sequel platform. From our genomic and comparative analyses, we drew three conclusions. First, compared with *M. perniciosa*, *Cladobotryum* (*C. dendroides* and *C. protrusum*) lost many repeated sequences over evolutionary time, which resulted in a contraction in the sizes of both genomes. Second, *C. dendroides* shared highly conserved syntenic blocks with *C. protrusum.* In addition, within the Hypocreaceae, *Cladobotryum* was more closely related to *Mycogone* than to *Trichoderma*. Third, a significant number of the predicted expanded gene families in *C. dendroides* were strongly associated with pathogenicity, virulence, resistance, and adaptation, providing insights into the molecular biology, genetics, evolution, and pathogenicity of this species. Overall, our genomic data provide a valuable resource with which to accelerate functional studies of the pathogen *C. dendroides*, subsequently supporting the development of control strategies as well as breeding programs designed to improve cobweb disease resistance in edible mushrooms.

## Figures and Tables

**Table 1:** Features of available *Cladobotryum* genomes.

Genome Features	*C. dendroides*	*C. protrusum*
Sequence coverage (X)	164	160
Genome size (Mb)	36.69	39.09
Total number of contigs	8	18
Contig N50 (bp)	4,995,562	4,973,539
Contig N90 (bp)	4,617,620	1,928,814
GC content (%)	48.20	47.84
Transposable elements (%)	4.37	2.59
Protein coding genes	9548	11,003
tRNAs	225	242
BUSCO estimates (%)	98.31	99.70

## Supplementary Materials

The following are available online at https://www.mdpi.com/2076-0817/9/3/232/s1, Table S1: Annotated pathogen–host interaction (PHI) genes in the *Cladobotryum dendroides* genome, Table S2: List of fungal virulence factor genes in the *Cladobotryum dendroides* genome, based on the database of fungal virulence factors (DFVF), Table S3: List of protease genes in the *Cladobotryum dendroides* genome, based on the MEROPS database, Figure S1: Mating-type loci (MAT) analysis of *Cladobotryum dendroides*. Genomic architecture of the MAT and flanking genes in *C. dendroides*. The arrows represent the orientation of the MAT1-1 genes APC5 (Anaphase-promoting complex subunit 5), APN2 (DNA lyase), CIA30 (Complex I intermediate-associated protein 30), and CoxVIa (Cytochrome c oxidase subunit VIa).
