# Supplementary figures and images for: Genomic Features of Cladobotryum dendroides, Which Causes Cobweb Disease in Edible Mushrooms, and Identification of Genes Related to Pathogenicity and Mycoparasitism
Source: Pathogens. 2020 Mar 20;9(3):232. doi: 10.3390/pathogens9030232 (PMC7157644; doi:10.3390/pathogens9030232)

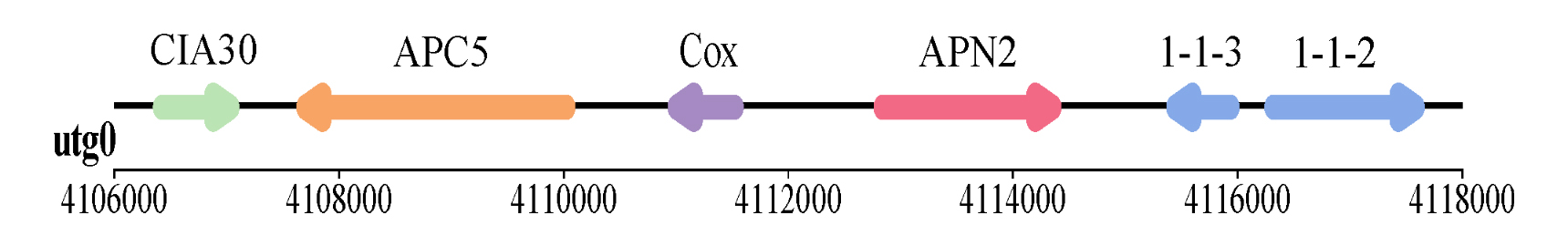

Supplement: Supplementary file 1 [file pathogens-09-00232-s001.zip › Supplementary File/Figure S1.jpeg]
